# Electronic Spectroscopy of YbNH_2_ and the Potential for Laser Cooling

**DOI:** 10.1021/acs.jpclett.5c00400

**Published:** 2025-03-25

**Authors:** Sophia Vadachkoria, Qingqing Lei, Timothy C. Steimle, Michael C. Heaven

**Affiliations:** † Department of Chemistry, Emory University, Atlanta, Georgia 30322, United States; ‡ Department of Chemistry, Arizona State University, Tempe, Arizona 85287, United States

## Abstract

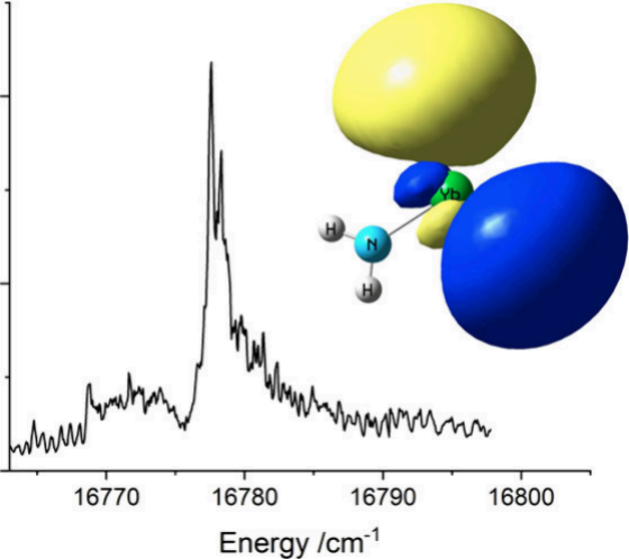

YbNH_2_ has been recognized as a suitable platform for observations of parity and time-reversal symmetry violations. Gas phase electronic spectra for YbNH_2_ were obtained for the *Ã*^2^*B*_2_-*X̃*^2^*A*_1_ and *B̃*^2^*B*_1_-*X̃*^2^*A*_1_ band systems. Both upper states are derived from the Yb^+^(4f^14^6s)NH_2_^–^ → Yb^+^(4f^14^6p)NH_2_^–^ electron promotion. Laser excitation and dispersed fluorescence measurements yielded band origins, vibrational constants and rotational band contours. Data for the *Ã*-*X̃* system show a Franck–Condon distribution that is favorable for laser cooling. An upper bound for the vibrational branching fraction for the 0_0_^0^ transition in emission was found to be 0.94, assuming negligible radiation to the low-energy Yb^+^(4f^13^6s^2^)NH_2_^–^ states. Quantum chemical calculations for the *Ã*–*X̃* vibronic bands are reported.

Here we report electronic spectra for gas phase ytterbium amide, YbNH_2_, which has been suggested as a nonlinear molecule to be used for searches of physics beyond the Standard Model (BSM).^1,2^ Heavy polar molecules are increasingly being used for BSM sensitive probes particularly in attempts to measure the electron electric dipole moment (eEDM), which can only be nonzero if both parity (P) and time-reversal (T) invariance are violated. The ^174^YbF isotopologue was the first molecular-based eEDM study.^3,4^ The relevant eEDM sensitive levels for the ^174^YbF isotopologue are the ^19^F(I = 1/2) hyperfine split components of the ground state: *X*^2^Σ^+^ (*N* = 0, *F* = 1 and 2). The experimental scheme for measuring the small energy level shift caused by a nonzero eEDM was based on a Ramsey interferometry method. The difference in phase for parallel and antiparallel orientation of static magnetic and electric fields is monitored.^5^ The sensitivity of the measurement is proportional to the product of the degree of polarization, *P(E*_*a*_*)*, the internal electric field of the molecule, *E*_int_, and the time it takes for the molecules to traverse the fields (coherence time), τ^6^. The degree of polarization, *P(E*_*a*_*)*, depends on the strength of the applied external static electric field *E*_*a*_. Significant improvements in the sensitivity of eEDM measurements may be accomplished by using other related Yb-containing molecules to increase *P(E*_*a*_*)* and/or the use of conditions that can lengthen τ. *E*_int_ for related Yb-containing molecules are expected to be similar to that of ^174^YbF (23.1 GV/cm).^7^ The previous molecular beam eEDM studies of ^174^YbF required large external electric fields (∼20 kV/cm) to achieve a *P(E*_*a*_*)* of 0.7^8^ due to the low polarizability of molecules in the *X*^2^Σ^+^ (*N* = 0, *F* = 1 and 2) levels. The required use of a high electric field can cause systematic errors due to current leakage and subsequent alternating magnetic fields.^9^

It was recognized that linear ^174^YbOH in its degenerate vibrational bending mode, which has nearly degenerate parity doublets, should be a more sensitive venue for eEDM measurements due in part to the increased polarizability.^10^ Subsequent theoretical calculations^11^ predicted that a *P(E*_*a*_*)* of 0.84 for ^174^YbOH can be achieve with an electric field strength of only 109 V/cm. It has also been suggested that the odd isotopologues ^173^YbF^12^ and ^173^YbOH^12,13^ are sensitive venues for new T,P-violating hadronic BSM physics via a measurement of the ^173^Yb nuclear magnetic quadrupole moment (NMQM) using an experimental scheme similar to that of the eEDM measurements. Again, the levels of the degenerate vibrational bending mode of ^173^YbOH should be more sensitive than those of the diatomic analogue due to the larger polarizability. A disadvantage of using parity doublet levels in a vibrational excited state of YbOH is the shorter spontaneous lifetime compared to the diatomic ground rotational states of YbF. Assuming a near-symmetric top *C*_2v_ structure, the levels of YbNH_2_ in the |*K*_*a*_| = 1 rotational manifold should also be closely spaced parity doublets that can be strongly polarized by the application of modest external electric fields. Furthermore, the relaxation of the |*K*_*a*_| = 1 levels down to the *K*_*a*_ = 0 manifold of levels is expected to be very slow. In addition to having no |*K*_*a*_| = 1 to *K*_*a*_ = 0 electric dipole transition moments, these transitions are forbidden, to a very good approximation, by nuclear state symmetries due to the equivalent protons. The |*K*_*a*_| = 1 levels are associated with **I**_T_ = 1 (ortho) and *K*_*a*_ = 0 levels with **I**_T_ = 0 (para). The nuclear spin statistics also will produce a favorable 3:1 non-Boltzmann population enhancement for the more energetic |*K*_*a*_| = 1 manifold under buffer gas cooling conditions.

A significant emphasis for enhancing the sensitivity of eEDM measurements has focused on using laser cooling.^6,9,14,15^ Laser cooling can significantly impact the sensitivity of eEDM measurements in two ways: slowing the forward velocity of the molecules to increase the coherence time, τ, and transverse cooling to collimate the molecular beam to increase the flux. The less demanding transverse laser cooling has been achieved for ^174^YbF^16^ and ^174^YbOH.^15^ The rotationally closed *X̃*^2^Σ^+^ (*N* = 1; *J* = 1/2, 3/2; *F* = 0, 1, 2) → *Ã*^2^Π_1/2_ (*J* = 1/2; *F* = 0, 1) transitions of ^174^YbF and ^174^YbOH were used for laser cooling. Then, for an eEDM measurement, population must be transferred into the relevant *X̃*^2^Σ^+^ (*N* = 0, *F* = 1 and 2) levels. To first approximation the *X̃*^2^Σ^+^ → *Ã*^2^Π_1/2_ transition of YbF and YbOH is a nonbonding Yb^+^(4f^14^,6s/6p_0_) to Yb^+^(4f^14^,6p_±1_/5d_±1_) promotion resulting in little change in the equilibrium structure. Consequently, the electronic transitions have nearly diagonal Franck–Condon factor (FCF) distributions, which is also a requirement for efficient laser cooling. The electronic structure and state distributions of YbF and YbOH share many of the characteristics with the extensively studied alkaline earth monovalent halides, hydroxides, and amides where the *X̃*^2^Σ^+^ → *Ã*^2^Π_1/2_ and *X̃*^2^*A*_1_ → *Ã*^2^*B*_2_, *B̃*^2^*B*_1_ transitions are M^+^(*n*s/*n*p_0_) to M^+^(*n*p_±1_/(*n* – 1)d_±1_) promotions of a nonbonding electron (hybrid atomic orbitals in parentheses).

It has been noted that the 1_11_ → 0_00_ (J_|K_a_||K_c_|_ notation) lines of the experimentally well characterized *X̃*^2^*A*_1_ → *Ã*^2^*B*_2_ electronic transitions of CaNH_2_, SrNH_2_, and BaNH_2_ are rotationally closed and provide a route for efficient laser cooling. It was speculated that there would be a similar transition in YbNH_2_.^1^ Recently, Frenett et al.^2^ performed a comparative study of the fluorescence branching ratios of SrNH_2_, SrOCH_3_, and SrSH and concluded that, from a branching ratio perspective, SrNH_2_ was more favorable for laser cooling than SrOCH_3_ and SrSH. It was suggested that the branching ratios for YbNH_2_ would also be conducive to laser cooling.

Direct laser cooling to slow the forward velocity of a molecular beam of heavy molecules is more challenging than transverse cooling and typically requires greater than 10^4^ photon scatters without significant loss to dark states. Unlike laser cooling and trapping of SrF,^17^ slowing and trapping YbF has proven to be much more challenging because of weak fluorescence decay from the *Ã*^2^Π_1/2_ state to low energy electronically excited states.^18,19^ The branching ratio for leakage from *Ã*^2^Π_1/2_ (Yb^+^(4f^14^,6s)F^–^(2p^6^)) to low-lying states derived from the Yb^+^(4f^13^,6s^2^)F^–^(2p^6^) configuration was predicted to be about 5 × 10^–4^, which is too large for effective radiation pressure slowing.^20^

Surprisingly, there are no published reports of spectroscopic or computational studies of YbNH_2_. In the present work we report an initial study of the *Ã*^2^*B*_2_-X̃^2^*A*_1_ band system, including radiative decay rate measurements and vibrational branching fractions for emission from the *Ã* state zero-point level. Electronic structure calculations were carried out to predict and facilitate the interpretation of the YbNH_2_ spectrum. Time-dependent density functional theory (TD-DFT) with B3LYP functionals was used to predict the properties of the 12 lowest energy states of YbNH_2_. The SARC-DKH-TZVPP basis set, including the SARC/J axillary functions was used for Yb.^21^ The cc-pVTZ basis sets were used for N and H.^22^ The spin–orbit operator was not included in these calculations, so we report Λ-Σ states.

The Orca 6.0 software package^23,24^ was used for these calculations. All states arising from the metal-centered 4f^14^6s, 4f^13^6s^2^ and 4f^14^6p configurations were considered. The *Ã*^2^*B*_2_, *B̃*^2^*B*_1_ and *C̃*^2^*A*_1_ states were associated with the 4f^14^6p configuration. Geometry optimization and vibrational frequency calculations were carried out for the *X̃*, *Ã*, *B̃* and *C̃* states. The results are collected in Table 1. For the vibrational normal modes we have adopted the numbering scheme for SrNH_2_ used by previous investigators.^2,25^ Mode 1 = N–H_2_ stretch (*a*_*1*_), 2 = NH_2_ bend (*a*_*1*_), 3 = Yb–N stretch (*a*_*1*_), 4 = YbNH_2_ out-of-plane bend (*b*_*1*_), 5 = NH_2_ asymmetric stretch (*b*_*2*_), and 6 = NH_2_ in plane wag (*b*_*2*_). The symmetry species are given in parentheses. In the following we label the vibronic transitions using the notation *M*_*vl*_^*vu*^, where *M* is the vibrational mode number, *vu* is the vibrational quantum number for mode *M* in the upper state and *vl* is the vibrational quantum number for the lower state. The optimized ground state structure yielded rotational constants of A = 12.95, B = 0.2179 and C = 0.2142 cm^–1^. Ray’s asymmetry parameter was −0.9993, showing that the molecule is very close to being a prolate top.

**Table 1:** YbNH_2_ Equilibrium Geometries (Å and Degrees), Electronic Term Energies (cm^–1^) and Harmonic Vibrational Constants (cm^–1^) from TD-DFT Calculations

	*X̃*	*Ã*	*B̃*	*C̃*
R (Yb–N)	2.2106	2.196	2.1967	2.2398
R (N–H)	1.0168	1.0162	1.0156	1.0161
θ (H–N–H)	104.99	105.02	105.2	104.9
T_e_	0	15053	15393	15942
ω_1_	3454	3467	3476	3463
ω_2_	1540	1536	1540	1543
ω_3_	473	472	472	445
ω_4_	433	437	392	366
ω_5_	3532	3540	3537	3538
ω_6_	293	283	286	336

The electronic state term energies from the TD-DFT calculations were sensitive to the choice of functional and basis set used for Yb. For example, the predicted *Ã*^2^*B*_2_-*X̃*^2^*A*_1_ transition energies ranged from 15080 cm^–1^ (B3LYP/SARC-DKH-TZVPP) to 19320 cm^–1^ (B3LYP/def2-TZVP/C). Rotational constants for the *X̃*^2^*A*_1_ and *Ã*^2^*B*_2_ states were estimated by comparison with SrNH_2_ and YbOH. Specifically, the H–N–H bond angles and N–H bond lengths for the *X̃*^2^*A*_1_ and *Ã*^2^*B*_2_ states were set equal to those of the determined for SrNH_2_^26^ and the Yb–N bond distances based on those determined for the *X*^2^Σ^+^ and *Ã*^2^Π_1/2_ states of YbOH^27^ scaled up by 7% in accordance with the observed increase in Sr–N bond length of SrNH_2_ as compared to the Sr–O bond lengths of SrOH. The resulting rotational constants from these structural estimates were: A = 12.79, B = 0.2235 and C = 0.2196 cm^–1^ for the *X̃*^2^*A*_1_ state and A = 12.79, B = 0.2307 and C = 0.2266 cm^–1^ for the *Ã*^2^*B*_2_ state.

Gas phase YbNH_2_ was generated by the reaction of laser ablated Yb with NH_3_. The products were cooled by free-jet expansion (see below). Oxides are always present with laser ablated metals, so we expected to see the YbNH_2_ spectrum accompanied by features arising from Yb, YbO and YbOH. The first vibronic band of YbNH_2_ was observed at 16776.8 cm^–1^.

The species assignment was based on the observations that this transition did not correspond to any of the known bands of YbOH or YbO and was only observed when NH_3_ was present in the carrier gas flow. Figure 1 shows a laser-induced fluorescence (LIF) scan over the 16776.8 cm^–1^ band. The laser-limited resolution of 0.3 cm^–1^ revealed the rotational contour but could not resolve the rotational structure. A dispersed laser-induced fluorescence (DLIF) spectrum obtained using excitation of the 16776.8 cm^–1^ band is shown in Figure 2. The short vibrational progression evident in this trace defined vibrational intervals of 470 and 463 cm^–1^, consistent with excitation of the Yb-NH_2_ stretch (cf. Table 1). The intensity distribution of this spectrum and the lack of any bands attributable to YbNH_2_ at lower excitation energies indicated that the 16776.8 cm^–1^ band was the origin of the *Ã*-*X̂* transition. An analysis of the peak intensities indicated that 94% of the emission returns population to the ground state zero-point level, under the assumption that radiative relaxation to the low-lying 4f^13^6s^2^ states is negligible (see below). Table 2 lists the bands of YbNH_2_ that were observed in this study.

**Figure 1:**
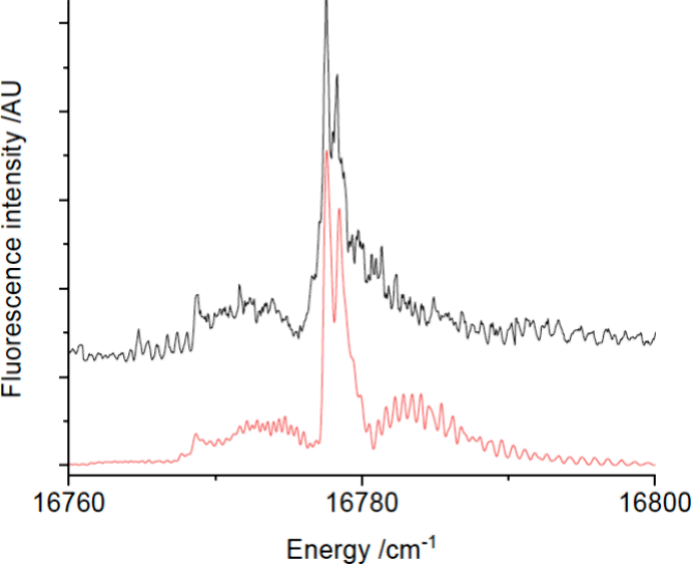
LIF scan of the YbNH_2_ band centered at 16776.8 cm^–1^. This is the K_a_′ = 0 ← K_a_″ = 1 sub-band. The lower trace is a simulation of the rotational contour generated by the PGOPHER software package. See text for details.

**Figure 2:**
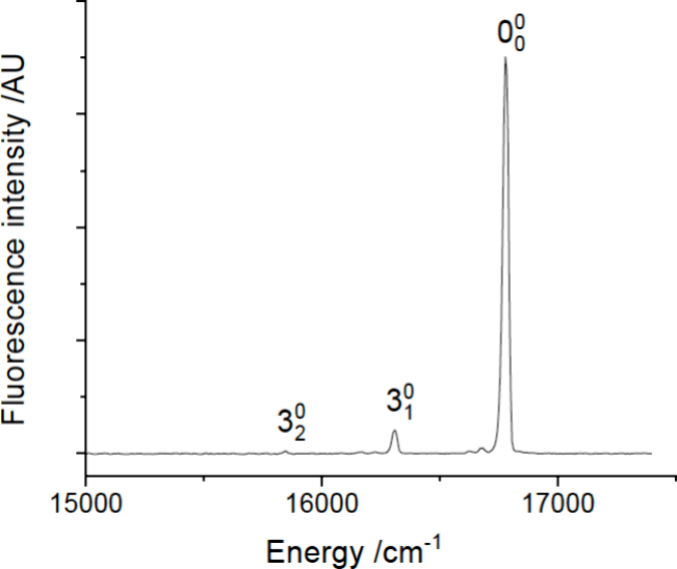
DLIF spectrum resulting from excitation of the 0_0_^0^ band at 16776.8 cm^–1^.

**Table 2:**
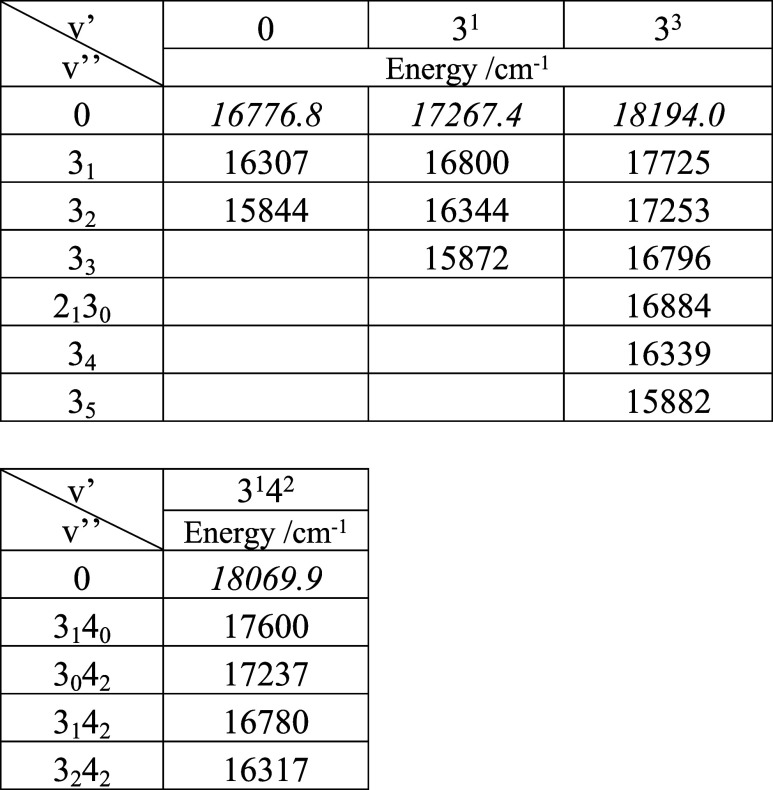
Band Centers Observed for the *Ã*-*X̃* Transition of YbNH_2_^a^

a Energies in cm^–1^ units. Error limits for values given in italics are ±0.1 cm^–1^. Error limits for values given in plain text are ±3 cm^–1^.

Modeling of the rotational intensity envelope of the 16776.8 cm^–1^ band was carried out using the PGOPHER software package.^28^ The ground state rotational constants from the electronic structure calculations were adopted and the ortho/para nuclear spin statistics were taken into account. The initial simulations assumed a *b*-type transition and were initiated with upper state rotational constants derived from the TD-DFT calculations (A′ = 12.86, B′ = 0.2204 and C′ = 0.2167 cm^–1^). Like CaNH_2_^29^ and SrNH_2_,^25^ it is expected that the *Ã*^2^*B*_2_ and *B̃*^2^*B*_1_ states will strongly interact producing a large second-order effective spin-rotation parameter, ϵ_*aa*_^(2)^, as well as a large second order contribution to the A-rotational parameter, *A*^(2)^. The second order perturbation theory expressions for these contributions are
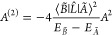
1and
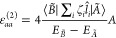
2where ∑_*i*_ζ_*i*_*l̂*_*i*_ is the sum of the product of the one electron orbital angular momentum and the associated spin–orbit parameter. Because the *Ã*^2^*B*_2_ and *B̃*^2^*B*_1_ states correlate to the *Ã*^2^Π state in a linear configuration ⟨*B̃*|*L̃*|*Ã*⟩ ≅ 1. In eq 1 “A” is the unperturbed rotational constant and the denominator is the energy interval between the *Ã* and *B̃* states. One uncertainty in using this model was that only one band was found that might be attributed to the *B̃*-*X̃* transition. As described below, this band was centered at 18120.5 cm^–1^, 1344 cm^–1^ above the zero-point level of the *Ã* state. Consequently, we have used this value to determine the energy difference. This yields a value of *A*^(2)^ = −0.5 cm^–1^ and an effective rotational constant of *A*_*eff*_ = 12.4 cm^–1^. It has been shown that for SrNH_2_ and CaNH_2_ that good agreement with the observed spin-rotation parameters can be realized if the ⟨*B̃*|∑_*i*_ζ_*i*_*l̂*_*i*_|*Ã*⟩ matrix element is taken as the spin–orbit parameter, *A*_so_, for the SrOH and CaOH, respectively. Although *A*_so_ has not been directly measured for YbOH, the Λ-doubling parameter experimentally determined for the *Ã*^2^Π_1/2_ state^27^ suggests a value of approximately 1270 cm^–1^ from which ϵ_*aa*_^(2)^ ≅ 50 cm^–1^.

Reducing the ϵ_*aa*_^(2)^ parameter to values around 40 cm^–1^ gave spectral predictions that were consistent with the shape of the origin band. Visual trial-and-error fitting of the band yielded the constants *A*_*eff*_′ = 11.2, B′ = 0.2219, C′ = 0.2181, ϵ_*aa*_^(2)^ = 41 cm^–1^ and an approximate rotational temperature of 65 K. The simulation in the region of the K_a_″ = 1 to K_a_′ = 0 sub-band is presented alongside the experimental spectrum is in Figure 1. The agreement is reasonably good given that the model only considers the ^174^Yb isotopologue (31.9% abundance, other significant isotopologues are ^172^Yb (21.8%), ^173^Yb (16.1%), ^171^Yb (14.2%), ^176^Yb (12.9%)). High resolution measurements are needed to refine the upper and lower state molecular constants, but the present modeling confirms that only small changes in the molecular geometry accompany *Ã*-*X̃* electronic excitation.

Scanning the excitation laser to shorter wavelengths revealed four additional vibronic bands of YbNH_2_. The band centers were located at 17267.5, 18069.9, 18120.5, and 18194.0 cm^–1^. These were interleaved with the bands of the YbOH *Ã*-*X̃* system which began at 17323 cm^–1^. Vibronic assignments for the higher energy bands of YbNH_2_ were primarily based on DLIF data. Figure 3 shows the DLIF spectrum resulting from excitation of the 17267.5 cm^–1^ band. This is a progression of the ground state Yb-NH_2_ stretch mode. The intensity distribution indicates that the upper state is 3^1^ with an *Ã* state fundamental vibrational interval of 490 cm^–1^.

**Figure 3:**
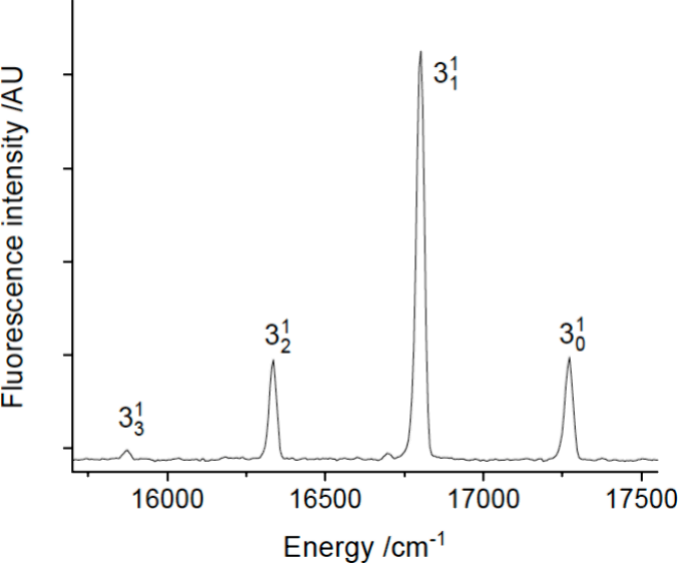
DLIF spectrum resulting from excitation of the 3_0_^1^ band at 17267.5 cm^–1^.

Figure 4 shows the DLIF spectrum obtained by exciting the 18069.9 cm^–1^ band. The peak assignments presented in this trace were consistent with the calculated ground state vibrational constants listed in Table 1. Note that vibrational mode 4, the out-of-plane bend, was only seen with even vibrational quanta as the motion transforms as the *b*_*1*_ representation.

**Figure 4:**
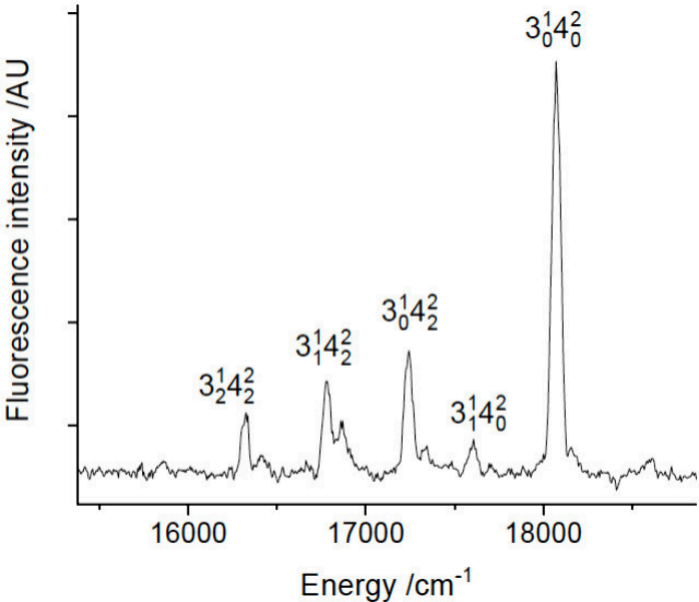
DLIF spectrum resulting from excitation of the 3_0_^1^4_0_^2^ band at 18069.9 cm^–1^.

The DLIF spectrum obtained by exciting the 18194.0 cm^–1^ band is shown in Figure 5. This is readily identified as a progression of the Yb-NH_2_ stretch resulting from excitation of the 3_0_^3^ transition. The weak band near 16684 cm^–1^ appears to be emission down to the *a*_*1*_ in-plane bending vibrational mode of the -NH_2_ moiety. The calculated harmonic frequency for 2_1_, 1540 cm^–1^ was close to the vibrational interval of 1510 cm^–1^ defined by the DLIF spectrum. Excitation spectra for the 3_0_^1^ and 3_0_^3^ transitions are reported here, but we were unable to obtain reliable data for the 3_0_^2^ transition as it was obscured by the 17730 cm^–1^ band of YbOH (the midpoint between YbNH_2_ 3_0_^1^ and 3_0_^3^ is 17730.8 cm^–1^).^30^ DLIF spectra excited via the 0_0_^0^, 3_0_^1^ and 3_0_^3^ bands exhibited progressions in the ground state Yb-NH_2_ stretch mode. Averaging these data yielded vibrational constants of ω_e_ = 475(2) and ω_e_x_e_ = 2.3(3) cm^–1^. Figure 4 shows a short progression of mode 3 built on the 4_2_ level. The average vibrational interval of 460 cm^–1^ indicates that excitation of the out-of-plane bend slightly reduces the Yb-NH_2_ vibrational frequency.

**Figure 5:**
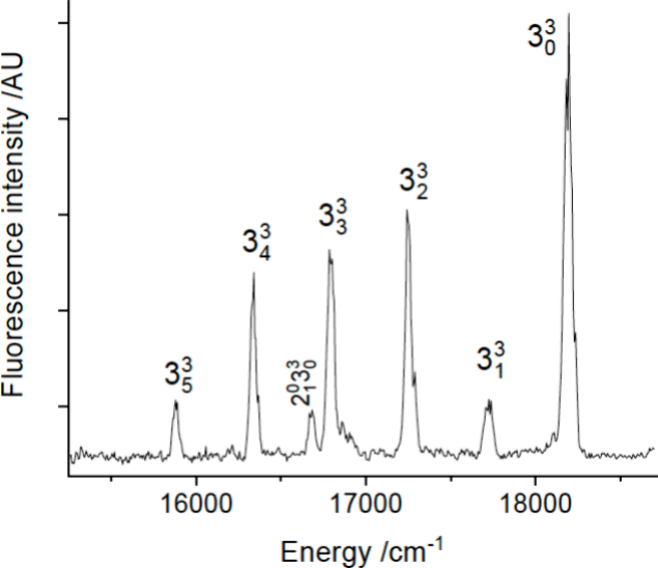
DLIF spectrum resulting from excitation of the 3_0_^3^ band at 18194.0 cm^–1^.

Fluorescence decay curves were recorded for the five observed upper states of YbNH_2_ and they were fitted to single exponential functions to determine the radiative lifetimes. As the decay times were comparable to the excitation laser pulse duration (nominally 10 ns) the results had large error ranges. They were consistent with an average lifetime of 50 ± 20 ns.

The DLIF spectrum for the 0_0_^0^ band was extended down to 13500 cm^–1^ to search for transitions down to low-energy states. The bands shown in Figure 2 were the only features detected in the 13500 – 16800 cm^–1^ range. However, this range was probably not sufficient for detection of the states arising from the 4f^13^6s^2^ configuration. Our TD-DFT calculations indicated that these states fall in the energy range of 4500–9000 cm^–1^, so that the highest energy transition radiating down from 0_0_^0^ would be near 12300 cm^–1^ where the sensitivity of our detection system was low. In future experiments it will be of value to search for the 4f^13^6s^2^ states using a near IR detection system and/or the excitation of higher energy transitions. Previously, we had observed the lowest energy 4f^13^6s^2^ states of YbF by exciting bands that were found in the near UV spectral range.^18^ This had the advantages that the emission bands were shifted to shorter wavelengths and the state mixing at higher energies increased the transition moments for relaxation to down to 4f^13^6s^2^ states. If UV bands of YbNH_2_ can be observed, they will facilitate the characterization of the 4f^13^6s^2^ manifold.

The 0_0_^0^ band DLIF spectrum (Figure 2) shows two weak features that are red-shifted by 100 and 152 cm^–1^ from the origin band. The same pair can be seen in association with the 3_1_^0^ emission. These satellite bands are too close to the parent transitions to be assigned to low-energy vibrational modes. It is probable that they arise from accidental excitation of populations trapped in vibrationally and/or |K_a_| excited levels that had not been fully relaxed by the jet-expansion process.

A surprising aspect of the present study is that we found only one band that could be attributed to the *B̃*-*X̃* or *C̃*-*X̃* transitions. This was centered at 18120.5 cm^–1^. Figure 6 shows the DLIF spectrum for this band, alongside the spectrum obtained by exciting the *Ã*-*X* 0_0_^0^ band (lower trace). The *x*-axis of Figure 6 is the red-shift relative to the excitation energy, so that bands to common lower state levels will be aligned. For trace (b) the amplitude of the *Ã*-*X̃* spectrum below −650 cm^–1^ has been multiplied by a factor of 10 to make the 3_2_^0^ band visible. The band intensity distribution for the spectrum that we tentatively identify as originating from the *B̃* state indicates emission from the zero-point level, but with somewhat more off-diagonal FCF contributions than the DLIF spectrum obtained by exciting the *Ã*-*X̃* 0_0_^0^ transition. We have not yet observed transitions that could be attributed to the *C̃*-*X̃* transition.

**Figure 6:**
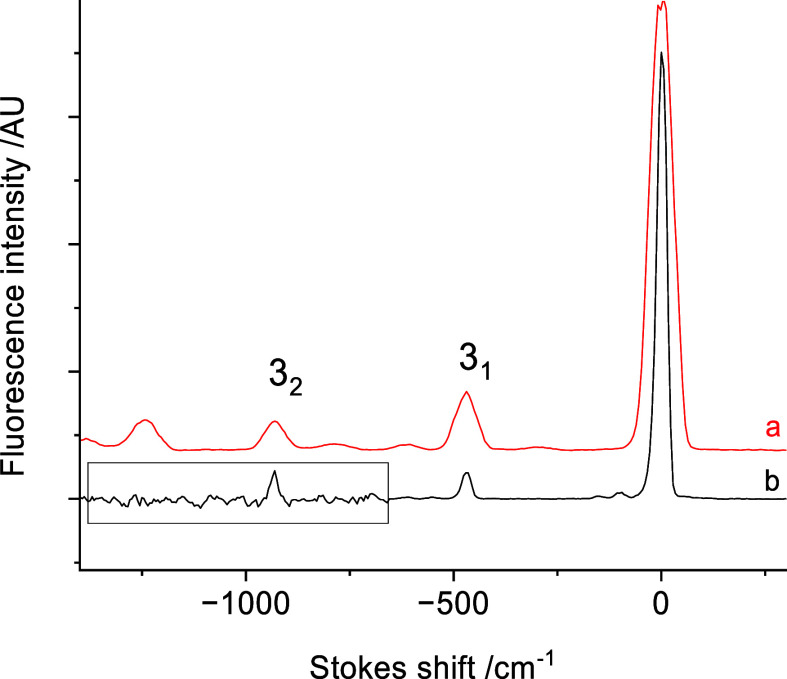
DLIF spectra for excitation of the band at 18120.5 cm^–1^ (trace a) and the *Ã*-*X̃* 0_0_^0^ band (trace b). The amplitude of trace b inside the box has been magnified by a factor of 10 to reveal the 3_2_^0^ band.

To summarize, the *Ã*^2^*B*_2_-*X̃*^2^*A*_1_ transition of YbNH_2_ has been characterized using LIF and DLIF techniques. A contour analysis of the 0_0_^0^ band, the associated DLIF spectrum and the predictions of electronic structure calculations indicate that excitation to the *Ã* state results in only small changes in the equilibrium geometry. The vibrational branching ratio for emission down to the ground state zero-point level was 94%. This should be regarded as an upper bound, as emission to states arising from the low-lying 4f^13^6s^2^ states was estimated to be outside the range of our detection system. However, these channels are likely to be minor contributors. For example, the emission of YbF A^2^Π_1/2_ v = 0 to the 4f^13^6s^2^ states was found to have a branching ratio^20^ of 5 × 10^–4^. Vibrationally excited levels of the *Ã* state were observed, with the active modes being the Yb-NH_2_ stretch and the out-of-plane bend. Vibrational intervals for both the *Ã* and *X̃* states were in acceptable agreement with the predictions of TD-DFT calculations. Overall, the data obtained in this study show that YbNH_2_ looks favorable for transverse laser cooling and that it is worthy of further investigations using high-resolution techniques.

## Experimental Methods

The LIF and DLIF techniques used in these experiments have been described previously.^18,31,32^ Briefly, a ytterbium (Yb) rod was ablated using a Q-switched Nd:YAG laser (Quanta-Ray DCR-1A, 10 mJ/pulse at 1064 nm) to vaporize Yb. For consistent ablation, the Yb rod was continuously rotated and translated. The vaporized Yb was entrained in a free-jet expansion driven by a 4% NH_3_/He gas mixture at a backing pressure of 82 psi. A pulsed valve (Parker-Hannifin Series 9) was used to supply the gas mixture. The pulse duration was 400 μs and the gas was expanded through a 3 mm diameter orifice to produce a supersonic free-jet. At 6 cm downstream from the nozzle orifice, the molecular beam was perpendicularly intercepted by the output of a dye laser (Lambda-Physik FL 3002e) pumped by a XeCl excimer laser (Lambda-Physik Complex Pro 201). The dye laser had a pulse duration of 10 ns and a FWHW line width of 0.3 cm^–1^. A portion of the dye laser output was sent through an iodine vapor cell to perform absolute wavenumber calibration by simultaneously recording the I_2_ B-X excitation spectrum with a photomultiplier tube (PMT, Hamamatsu R943-02).

For the LIF measurements, the fluorescence emitted by the molecular beam was collimated and focused onto a PMT (Hamamatsu R955). Long-pass filters with a cut-on wavelength approximately 20 nm longer than the dye laser wavelength were positioned in front of the PMT to reduce laser scatter. The PMT signal was sent to a digital oscilloscope (Agilent Technologies DSO1024A) and a boxcar integrator (Stanford Research Systems SR250). For the lifetime measurements, fluorescence decay curves were obtained by averaging the signal from 256 laser pulses per curve and downloading the data directly from the oscilloscope. Background measurements, also averaged over 256 pulses, were recorded without the ablation laser. The difference between the signal and background measurements was calculated and averaged to eliminate chemiluminescence and scatted laser light from the signals.

For the DLIF measurements, spectra were recorded using a 0.64 m monochromator with a 1200 grooves/mm diffraction grating (ISA HR640), replacing the long-pass filter. The relative intensities of the DLIF spectra were corrected to account for the wavelength-dependent spectral response of the monochromator/PMT combination. This calibration was performed using a tungsten filament light bulb, assuming that Planck’s blackbody radiation model could reasonably approximate its radiation. The filament temperature, approximately 1720 K, was determined using an optical pyrometer (Capintec Instruments Hot-Shot, ROS-5). After applying the response correction to the DLIF spectrum, the relative intensity of each spectral band was calculated by dividing its intensity by the sum of the intensities of all bands.

## Supporting Information Available

Transparent Peer Review report available (PDF)
