# Supplementary material for: Electronic Spectroscopy of YbNH2 and the Potential for Laser Cooling
Source: J Phys Chem Lett. 2025 Mar 25;16(13):3309–15. doi: 10.1021/acs.jpclett.5c00400 (PMC11973910; doi:10.1021/acs.jpclett.5c00400)
Supplement: Supplementary file 1 — jz5c00400_si_001.pdf [file jz5c00400_si_001.pdf]

jz-2025-00400t.R1

Name: Peer Review Information for "Electronic spectroscopy of YbNH₂ and the potential for laser cooling"

First Round of Reviewer Comments

Reviewer: 1

Comments to the Author

Accurately characterizing the vibronic transitions of YbNH2 is timely as this could be a useful molecule in the quest for observation of the eEDM magnitude as predicted by different beyond-standard-model physics models. The transitions observed experimentally in this paper and characterized with the aid of TDDFT modeling are relevant for this purpose, although characterization of also the lower lying f13 states may be needed to fully assess the feasibility of laser cooling for this molecule.

The currently presented data will be very useful for validating higher level theoretical approaches that can potentially assess also the f13 states . After these f13 states are characterized (either experimentally or theoretically) the feasibility of laser cooling and eEDM experiments can be assessed.

The paper need little modification, but I did spot two minor errors (points 1 and 2 below) to correct as well as an unclear formulation (point 3) that should be improved upon.

1) page 4, line 16

An artefact ("Click here..|) of formatting the paper should be removed,

2) page 4, lines 21-22

The designation of F[2p6] as F+ is obviously wrong. Should be F-.

3) page 5, line 46. "An empirical scaling based on the electron affinities of the ligands "

The precise way in which the empirical scaling is performed as well as the error bars relating to fitting a specific functional form to experimental data are not provided. This makes this statement useless. I'd recommend to either report this procedure properly or show the spread of energies obtained by different DFT functionals to provide an indication of the reliability of the calculations.

Reviewer: 2

Comments to the Author

I have been asked to review the manuscript entitled ‘Electronic spectroscopy of YbNH2 and the potential for laser

cooling’.

Overall, I find this manuscript exceptionally well suited for JPCL after the authors have accounted for the following minor issues. It does not have to be reviewed again.

Abstract: the abstract shall be a summary of the results – I do not see that. Please provide a meaningful abstract.

Page 4: delete: ‘Click or tap here to enter text’

Table 1: add units in caption

Author's Response to Peer Review Comments:

We thank the reviewers for their helpful suggestions, and we have made all of the recommended changes. Our responses are given in red text below:

Reviewer: 1

Recommendation: This paper is publishable subject to minor revisions noted. Further review is not needed.

Comments:
Accurately characterizing the vibronic transitions of YbNH2 is timely as this could be a useful molecule in the quest for observation of the eEDM magnitude as predicted by different beyond-standard-model physics models. The transitions observed experimentally in this paper and characterized with the aid of TDDFT modeling are relevant for this purpose, although characterization of also the lower lying f13 states may be needed to fully assess the feasibility of laser cooling for this molecule.

The currently presented data will be very useful for validating higher level theoretical approaches that can potentially assess also the f13 states. After these f13 states are characterized (either experimentally or theoretically) the feasibility of laser cooling and eEDM experiments can be assessed.

The paper need little modification, but I did spot two minor errors (points 1 and 2 below) to correct as well as an unclear formulation (point 3) that should be improved upon.

1) page 4, line 16

An artefact ("Click here..|) of formatting the paper should be removed,

This text has been deleted

2) page 4, lines 21-22

The designation of F[2p6] as F+ is obviously wrong. Should be F-.

The charge has been corrected

3) page 5, line 46. "An empirical scaling based on the electron affinities of the ligands "

The precise way in which the empirical scaling is performed as well as the error bars relating to fitting a specific functional form to experimental data are not provided. This makes this statement useless. I'd recommend to either report this procedure properly or show the spread of energies obtained by different DFT functionals to provide an indication of the reliability of the calculations.

We have removed the comment on the empirical scaling and noted the range of transition energies obtained from our TD-DFT calculations.
(New text immediately below Table 1)

Reviewer: 2

Recommendation: This paper is publishable subject to minor revisions noted. Further review is not needed.

Comments:
I have been asked to review the manuscript entitled ‘Electronic spectroscopy of YbNH2 and the

potential for laser cooling'.

Overall, I find this manuscript exceptionally well suited for JPCL after the authors have accounted for the following minor issues. It does not have to be reviewed again.

Abstract: the abstract shall be a summary of the results – I do not see that. Please provide a meaningful abstract.

The abstract has been revised to be a better summary of the results

Page 4: delete: 'Click or tap here to enter text'

This text has been deleted

Table 1: add units in caption

The units have been added to the caption of Table 1
